# Clinical Trial and *In Vitro* Study for the Role of Cartilage and Synovia in Acute Articular Infection

**DOI:** 10.1155/2015/430324

**Published:** 2015-11-10

**Authors:** Elia R. Langenmair, Eva J. Kubosch, Gian M. Salzmann, Samuel Beck, Hagen Schmal

**Affiliations:** ^1^Department of Orthopedics and Trauma Surgery, Albert-Ludwigs University Medical Center Freiburg, Hugstetter Street 55, 79106 Freiburg, Germany; ^2^Schulthess Clinic, Zurich, Lenghalde 2, 8008 Zurich, Switzerland; ^3^Department of Orthopaedics and Traumatology, Odense University Hospital and Department of Clinical Research, University of Southern Denmark, Sdr. Boulevard 29, 5000 Odense C, Denmark

## Abstract

*Objective*. Osteoarthritis is a long-term complication of acute articular infections. However, the roles of cartilage and synovia in this process are not yet fully understood. *Methods*. Patients with acute joint infections were enrolled in a prospective clinical trial and the cytokine composition of effusions compared in patients with arthroplasty (*n* = 8) or with intact joints (*n* = 67). Cytokines and cell function were also analyzed using a human *in vitro* model of joint infection. *Results*. Synovial IL-1*β* levels were significantly higher in patients with arthroplasty (*p* = 0.004). Higher IL-1*β* concentrations were also found in the *in vitro* model without chondrocytes (*p* < 0.05). The anti-inflammatory cytokines IL-4 and IL-10 were consistently expressed *in vivo* and *in vitro*, showing no association with the presence of cartilage or chondrocytes. In contrast, FasL levels increased steadily *in vitro*, reaching higher levels without chondrocytes (*p* < 0.05). Likewise, the viability of synovial fibroblasts (SFB) during infection was higher in the presence of chondrocytes. The cartilage-metabolism markers aggrecan and bFGF were at higher concentrations in intact joints, but also synthesized by SFB. *Conclusions*. Our data suggest an anti-inflammatory effect of cartilage associated with the SFBs' increased resistance to infections, which displayed the ability to effectively synthesize cartilage metabolites.The trial is registered with DRKS 00003536, MISSinG.

## 1. Introduction

Although the treatment of synovial bacterial infections usually succeeds, long-term complications include the development of osteoarthritis (OA) [1]. Proinflammatory mediators are suspected to influence chondrocyte differentiation and survival. The role of chondrocytes in this process is not yet fully understood; however, there is growing evidence that cartilage plays an active role during inflammation and is not only target [2]. Furthermore, the interaction between synovia and chondrocytes seems to contribute to the balance of anabolic and catabolic processes associated with OA. Considering the capacity of synovia to regenerate and cartilage's limited capacity to do so, the relation between these two anatomical structures might be decisive in regulating repair processes [3]. The approach taken in this study is a combination of biomarker analysis done in patients with acute synovial infections and a previously described* in vitro* model for intra-articular infections [4]. To discriminate between cartilage effects, we compared patients with an intact joint to patients having a total endoprosthesis. In the latter group, the effects of cartilage on inflammatory processes are eliminated. This situation was simulated* in vitro* comparing the articular infection model in a setting with or without chondrocytes. Our analysis included aggrecan, an essential component of the extracellular matrix and a sensitive marker for OA progress. Furthermore, bFGF, a typical cartilage metabolite associated with repair processes, was assessed [5]. We measured IL-1*β* to characterize the degree of inflammation [6]. Inspired by evidence of cartilage's anti-inflammatory properties, we also investigated the role of cytokines such as IL-4, IL-10, and Fas ligand (FasL) playing a potential role in our models and* in vivo*. Furthermore, TGF*β* levels were assessed dependent on the presence of chondrocytes. We hypothesized that cartilage would reveal immunomodulatory features and similar* in vivo* and* in vitro* patterns.

## 2. Material and Methods

### 2.1. Clinical Trial

As has already been published [4], a consecutive series of 75 patients treated between April 2011 and November 2012 presenting the clinical symptoms of bacterial joint infection were recruited for the prospective collection of joint fluid. All patients suffered from pain, swelling, effusion, and elevated inflammatory serological parameters (e.g., C-reactive protein). Effusion from 76 affected joints was included in the analysis. Infections of knee (75%), hip (6.6%), ankle (1.3%), and shoulder (17.1%) were included. The trial was registered (MISSinG, DRKS 00003536) and approved by the Ethics Board of the University of Freiburg (AN-EK-FRBRG-50/11). All patients participating in this study provided their written consent. There were 4 other patients whom we intended to include, but they had to be excluded because of storage-protocol violation (*n* = 3) or sudden death because of fulminant lung embolism (*n* = 1, no written consent). Effusion was taken during arthroscopy or preoperative puncture and immediately frozen. Specimens were stored in liquid nitrogen until analysis. 81% of isolated bacteria belonged to* Staphylococcus* species. Evaluation via the Kellgren-Lawrence-Score [7] based on conventional X-rays was done by 3 independent orthopedic surgeons (EL, EJK, and HS), resulting in a consensus decision.

### 2.2. *In Vitro* Model

We employed the* in vitro* model of synovial infection as previously described [4]. Briefly, human chondrocytes (CHDR) were isolated from femoral heads with a Kellgren-Lawrence-Score ≤ 2 obtained during hip arthroplasty following femoral neck fractures. Following collagenase digestion, cells were cultured in Ham's F-12 medium with 1% FBS (Invitrogen, Karlsruhe, Germany) and 1% penicillin/streptomycin (Invitrogen, Karlsruhe, Germany). Human synovial fibroblasts (SFB) were isolated from synovia biopsies obtained during knee operations with an arthrotomy applying a similar protocol involving collagenase digestion and using the same media composition as indicated for CHDR. Human peripheral blood mononuclear cells (MNC) were isolated from heparin-treated human whole blood by a Ficoll-Paque (Pharmacia, Piscataway, NJ, USA) gradient. For 3D coculture, CHDR were embedded into alginate beads. Then, the cells were assembled having the SFB in monolayer overlayed with the CHDR in alginate beads; MNC were separated in the same well by cell culture inserts with 1 *μ*m pore size (BD, Heidelberg, Germany). After 3-day preconditioning, the coculture was challenged with 250 ng/mL LPS (Product number L6529, serotype* E. coli* 055:B5, Sigma Taufkirchen, Germany) and/or 100 ng/mL PMA (Product number P1585, Sigma Taufkirchen, Germany). Similar to previously reported data [4], the inflammatory reaction induced by LPS alone was limited and insufficient to reveal differential influences of the absence of cartilage. PMA alone had significant but fewer proinflammatory effects than the LPS and PMA combination; therefore, that data is not reported. Induction of inflammation by the significant overexpression of IL-1*β* was verified in each experiment. On days 3 and 7, half of the media were exchanged to reduce the inflammatory burden mimicking a staged lavage as applied to treat* in vivo* joint infections. Media collected on days 0, 3, 7, and 10 were preserved at −80°C for analysis. Cocultures containing CHDR in alginate beads or not were compared in order to simulate joints with and without (arthroplasty) cartilage. The cells' preparation protocols were approved by the Ethics Committee of the University of Freiburg as part of the ‘Tissue Bank for Research in the Field of Tissue Engineering” project (GTE-2002) and the biobank “Osteo” (AN-EK-FRBRG-135/14). Cells from the same donors were used when comparing different culture conditions. Lipopolysaccharides (LPS) are a cell-wall component of Gram-negative bacteria and provoke a distinct inflammatory stimulus including a potent activation of the macrophage secretory reaction [8]. While the LPS response is quickly diluted, a phorbol 12-myristate 13-acetate (PMA) application leads to stable, nontransient IL-1 accumulation [9]. Therefore, LPS and PMA are ideal substances to modulate the period and severity of an* in vitro* inflammation.

### 2.3. ELISA and Protein Content

Cytokine and protein concentrations (interleukin- [IL-] 1*β*, IL-4, IL-10, aggrecan [ACAN], basic fibroblast growth factor [bFGF], and Fas ligand [FasL]) and transforming growth factor [TGF]*β* in synovial joint fluids from acutely infected knee joints or coculture supernatants were analyzed by ELISA (RnD, Minneapolis, MN, USA, and BioSource Deutschland GmbH, Solingen, Germany) according to the manufacturers' instructions. Briefly, this assay employs the quantitative sandwich enzyme immunoassay technique. The microplate was precoated with a specific monoclonal antibody. Supernatants were applied to the wells and, after washing, an HRP-conjugated specific antibody was added to the wells. Following the next wash, color development was proportional to the protein concentration and calculated by comparison with a standard. A colorimetric method was applied to quantify total protein amount in the lavage fluids. The bicinchoninic acid (BCA) assay was available in kit form from Pierce (Rockford, IL, USA) and used according to the manufacturer's instructions [4]. All data from the analyzed cytokines and proteins are reported as relative expression to the total protein content. Statistical calculations were based on these values.

### 2.4. CellTiter 96 AQueous One Solution Cell Proliferation Assay (MTS Assay)

Reagents were provided by Promega (Madison, WI, USA) and used according to the manufacturer's instructions [5]. MTS tetrazolium is bioreduced by cells into a colored formazan product that is soluble in tissue culture medium. Following a 10-day growth period, the reagent was added to each well of a microtiter plate. As per the manufacturer's instructions, we observed optical density at 490 nm after a 4-h incubation period.

### 2.5. Histology of SFB

SFB grown on cover slides in coculture approaches were fixed by PBS buffered 2% formaldehyde for 24 h at room temperature, followed by Giemsa staining for 10 min. Image acquisition and analysis were done by Zeiss Axioplan 200 and Axiovision 4.6, respectively [4].

### 2.6. Real Time PCR (RT-PCR)

RNA samples from days 7 and 14 were transcribed into cDNA; RNA analysis was carried out for gene expression of TGF*β*. Total mRNA was prepared using the Qiagen RNeasy kit according to manufacturer's instructions (Qiagen, Hilden, Germany). Total RNA (1 *μ*g) was treated with 1 U DNAse I (Invitrogen, Karlsruhe, Germany) to remove genomic DNA. Poly-T primed cDNA synthesis was done using 1 U reverse transcriptase III (RTIII, Invitrogen) per 1 *μ*g RNA according to manufacturer's instruction. TaqMan PCR assays were performed in 384-well plates in Roche LightCycler480 (Roche, Mannheim, Germany) using the Roche LightCycler Mastermix. For gene expression analyses, Roche's universal ProbeLibrary Probes and recommended Universal ProbeLibrary Reference Gene Assays were used. The 2-step cycling conditions were denaturation: 95°C for 5 min, followed by 45 amplification cycles: 95°C, 10 sec, 60°C, 35 sec, and 72°C, 1 sec. Data was quantified via ΔΔCT comparisons. Data were normalized by comparing genes of interest versus reference genes (GAPDH). Reaction efficiency is controlled by a relative standard curve and/or a calibrator per reaction. RT-PCR was done in triplicate; each value represents an average of 4 experimental preparations.

### 2.7. Data Analysis and Statistics

Concentrations of proteins and cytokines determined by the specific ELISAs and the BCA assay were calculated according to the manufacturers' instructions (RnD, Minneapolis, MN, USA; Thermo Scientific, Rockford, IL, USA), creating a standard curve and reducing data using a four-parameter logistic (4-PL) curve fit by using GraphPad Prism 5 software (GraphPad Software, Inc., La Jolla, CA, USA). All values were expressed as mean ± standard error of the mean. Regarding the scores and all numerical values, statistical significance was tested nonparametrically primarily using the* U*-test according to Mann and Whitney. Numeric data sets were then further examined with one- and two-way analysis of variance, and individual group means were compared with the unpaired Student's* t*-test. In case of unequal variances, we applied the Aspin-Welch test. Incidences were compared using the Chi square test. Statistical significance was defined as *p* < 0.05.

## 3. Results

### 3.1. Characterization of Patients

75 patients with 76 articular infections were included in a prospective clinical trial. We compared the group with endoprostheses (*n* = 8) to the group with a normal joint (*n* = 68). Patients in both groups differed significantly according to certain clinical and epidemiological characteristics as shown in Table 1. In short, the postarthroplasty patients were older and presented more comorbidities and their diseases were more serious. The mean Kellgren-Lawrence-Score of the joints without endoprostheses was 1.6 ± 0.26, reflecting a mean moderate state of joint degeneration. In order to gain collectives with better comparability, patients with exclusive knee arthroplasties were separately analyzed (*n* = 6/51, Table 1). Although again clinical signs of disease were more serious, there was no statistically significant difference in age and the portion of isolated* Staphylococcus* species increased to 86% in this subgroup.

### 3.2. Total Protein Content

We detected no statistically significant difference in the total protein concentration in the inflamed joints with or without endoprosthesis (0.057 ± 0.014 g/mL versus 0.048 ± 0.003 g/mL). This was also apparent* in vitro* (0.00178 ± 2.85  ×  10^−5^ g/mL versus 0.00161 ± 1.38  ×  10^−4^ g/mL) with no substantial variance during the culture period.

### 3.3. IL-1*β*

**Figure 1:**
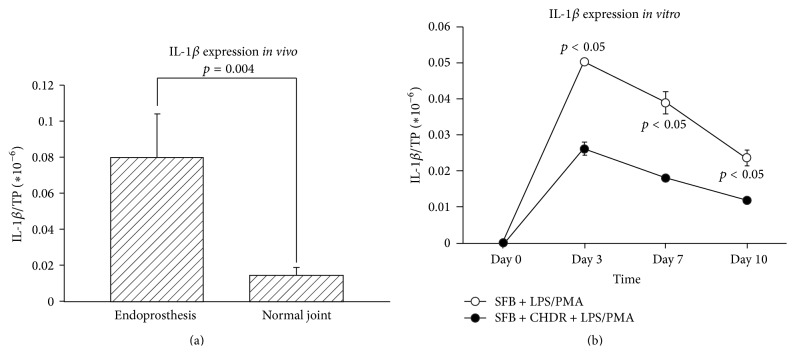
(a) shows that synovial IL-1*β* levels were statistically significantly higher in patients with an endoprosthetic implant (no cartilage) than in those with normal joints (with cartilage). (b) demonstrates the* in vitro* model's corresponding data. Similarly, IL-1*β* concentrations were statistically significantly higher in the setting without chondrocytes.

According to calculations of all our patients' data, synovial IL-1*β* levels correlated statistically significantly with initial serological CRP concentrations (Spearman correlation coefficient 0.3, *p* = 0.007). The group of patients with endoprostheses revealed higher IL-1*β* levels than those with normal joints (0.079 ± 0.025  ×  10^−6^ versus 0.015 ± 0.004  ×  10^−6^, *p* = 0.004,* U*-test, Figure 1(a)). This could be confirmed in the subgroup analysis of knee infections (0.087 ± 0.033  ×  10^−6^ versus 0.013 ± 0.005  ×  10^−6^, *p* = 0.02). This was also apparent* in vitro*; values peaked on day 3 in both groups (0.050 ± 0.001  ×  10^−6^ versus 0.026 ± 0.002  ×  10^−6^), declining thereafter. At all three measured time points after day 0, IL-1*β* was at higher concentrations in the cocultures without chondrocytes (*n* = 3 per group, *p* = 0.0495 at all time points,* U*-test, Figure 1(b)), demonstrating IL-1*β* induction via the inflammatory stimulus.

### 3.4. Aggrecan

**Figure 2:**
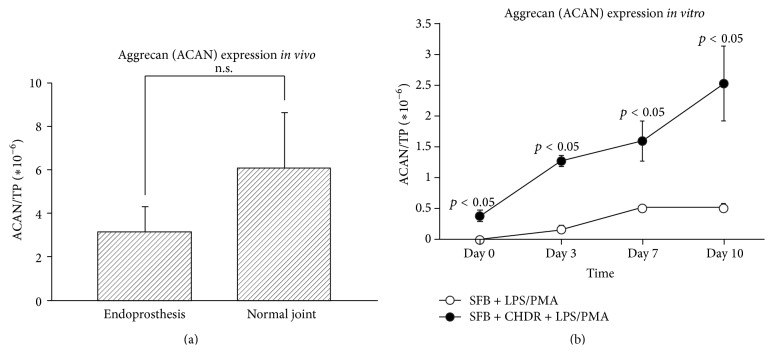
(a) shows that synovial aggrecan levels were reduced in patients with an endoprosthetic implant (no cartilage) compared to those with normal joints (with cartilage). This difference failed to attain statistical significance. (b) demonstrates the* in vitro* model's corresponding data. Similarly, aggrecan concentrations were statistically significantly higher in the setting with chondrocytes.

In contrast to IL-1*β*, the patient group with endoprostheses showed aggrecan levels that were lower than those with normal joints, although without attaining statistical significance (3.13 ± 1.14 × 10^−6^ versus 6.06 ± 2.57 × 10^−6^, *p* = 0.299, Figure 2(a)). This was found for all patients and in the subgroup of knee infections. This was also shown* in vitro*; values increased over time in both groups, reaching the highest values on day 10 (0.52 ± 0.06  ×  10^−6^ versus 2.52 ± 0.6  ×  10^−6^). At all four measured time points, aggrecan was measured at higher concentrations in the cocultures with chondrocytes (*n* = 3 per group, *p* < 0.05 for all time points,* U*-test, Figure 2(b)), demonstrating aggrecan release beginning with the preculture period.

### 3.5. bFGF

**Figure 3:**
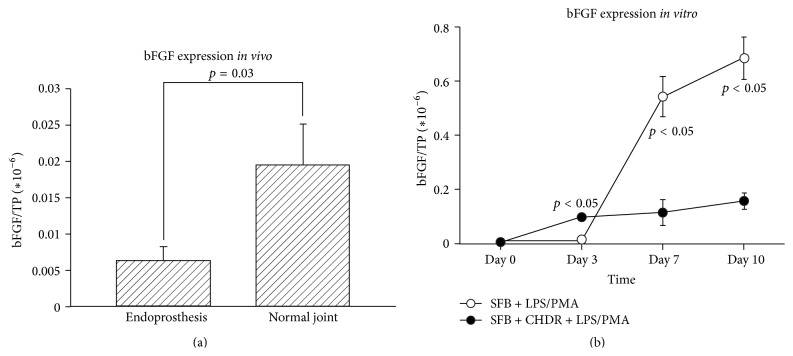
(a) shows that synovial bFGF levels were diminished in patients with an endoprosthetic implant (no cartilage) compared to those with normal joints (with cartilage). This difference reached statistical significance. (b) demonstrates the* in vitro* model's corresponding data; here, time-dependent regulation patterns revealed increasing bFGF concentrations in all groups over time. bFGF levels were higher in the group with CHDR on day 3, but lower on days 7 and 10.

bFGF concentrations were statistically significantly higher in the infected intact joints (0.0063 ± 0.0019  ×  10^−6^ versus 0.0195 ± 0.0057  ×  10^−6^, *p* = 0.031 [Aspin-Welch test], Figure 3(a), heterogeneous variances [*F*-Test 3.6  ×  10^−6^]). Although data of the subgroup with knee infections showed the same tendency, statistical analysis failed to confirm the significance of difference (0.0063 ± 0.0025  ×  10^−6^ versus 0.0190 ± 0.0072  ×  10^−6^, n.s.). The* in vitro* data showed a time-dependent expression pattern for bFGF, with higher levels in the coculture with chondrocytes on day 3 (0.0145 ± 0.0014  ×  10^−6^ versus 0.0995 ± 0.0111  ×  10^−6^, *p* = 0.0495). We observed a reversal on day 7, when the group without CHDR displayed higher levels (0.54 ± 0.07  ×  10^−6^ versus 0.12 ± 0.05  ×  10^−6^, *p* = 0.0495,* U*-test, Figure 3(b)).

### 3.6. Anti-Inflammatory Mechanisms

**Figure 4:**
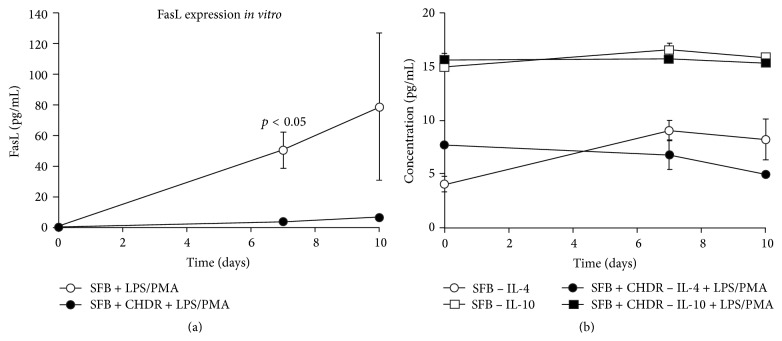
(a) shows FasL levels in the* in vitro* coculture. Concentrations increased over time in both groups but were statistically significantly more elevated in the group without CHDR. (b) illustrates IL-4 and IL-10 levels. We observed no dependency on time or presence of CHDR.

IL-4, IL10, and FasL were examined for a possible role as anti-inflammatory cytokines. Although we detected all these proteins, none were differentially regulated in joints with or without endoprostheses (IL-4/TP 1.19 ± 0.42  ×  10^−7^ versus 1.76 ± 0.62  ×  10^−7^, IL-10/TP 2.4 ± 0.89  ×  10^−4^ versus 4.8 ± 2.6  ×  10^−4^, and FasL/TP 9.8 ± 5.3  ×  10^−7^ versus 15.6 ± 2.15  × 10^−7^), which was also shown for the subgroup of knee infections. IL-4 and IL-10 also demonstrated this* in vitro*, showing no significant differences at the given time points, and higher absolute IL-10 values than those noted* in vivo* for IL-4 (Figure 4(b)). FasL (Figure 4(a)) exhibited consistently rising levels in both groups, reaching higher concentrations in the coculture without CHDR on day 7 (50.3 ± 11.8 pg/mL versus 3.55 ± 1.817 pg/mL, *p* = 0.0495) and day 10 (78.3 ± 48.3 versus 5.96 ± 0.979, n.s.).

### 3.7. SFB and CHDR Viability

**Figure 5:**
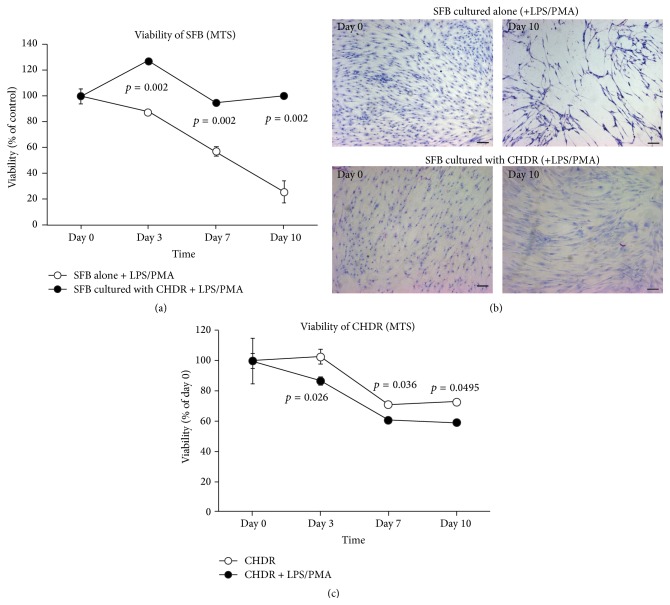
Viability of SFB was monitored using an MTS assay as shown in (a). While viability did not change over time in the presence of CHDR, a gradual decrease was observed during the incubation period in the absence of CHDR. (b) shows the correlating Giemsa staining for SFB cultures on cover slides confirming the MTS data. Scale bar 50 *μ*m. The results of the MTS assay for CHDR in alginate beads are shown in (c). Metabolic activity slightly decreased from day 7 and was enhanced by adding LPS and PMA.

To evaluate the viability of SFB, we carried out an MTS assay. While SFB cultured in the presence of CHDR demonstrated the same viability over the whole culture period under inflammatory conditions, SFB numbers decreased gradually in the absence of CHDR, falling to 25.8 ± 8.5% (Figure 5(a)). We confirmed this macroscopically via Giemsa staining for SFB cultures on cover slides (Figure 5(b)). During the coculture period, the metabolic activity of chondrocytes decreased slightly from day 7 to 72.8 ± 0.4% on day 10, a phenomenon that was slightly enhanced by adding LPS and PMA (59.2 ± 0.2%, Figure 5(c)).

### 3.8. TGF*β*

**Figure 6:**
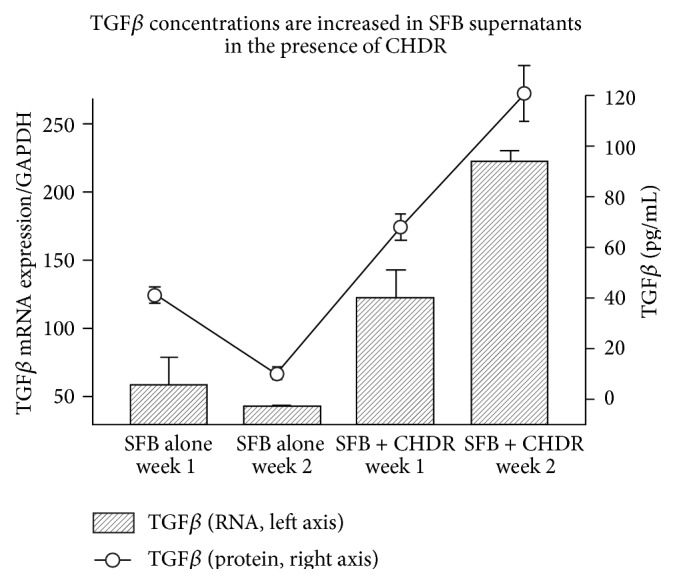
The coculture of SFB with CHDR caused an upregulation of both TGF*β* protein and TGF*β* mRNA (*p* < 0.05 for both time points) compared to SFB alone.

The amount of TGF*β* mRNA and protein was dependent on the presence of CHDR. The coculture with CHDR caused an upregulation of both TGF*β* protein in the supernatant (10.3 ± 1.8 SFB alone to 121 ± 11 pg/mL with CHDR after 2 weeks) and TGF*β* mRNA measured from SFB (43.5 ± 2.0-fold SFB alone to 221 ± 8.5-fold TGF*β* mRNA expression/GAPDH with CHDR after 2 weeks) when compared to SFB alone (Figure 6, *p* < 0.05 for protein and mRNA for both time points).

## 4. Discussion

This study's main finding is that cartilage exhibits anti-inflammatory properties during acute articular infections, as demonstrated by decreased IL-1*β* expression in the presence of cartilage or CHDR* in vivo* and* in vitro*. Although CHDR increased the resistance of SFB to* in vitro* inflammatory conditions, the clinical trial's data does not allow definitive conclusions about the mechanisms behind the anti-inflammatory effect.* In vitro* data suggest that FasL plays a key role, as it is in contrast to IL-4 and IL-10 increasingly secreted in the coculture during the inflammatory process. Furthermore, our results support the SFBs' capacity to synthesize typical representatives of cartilage metabolism such as aggrecan and bFGF. The production of these proteins was enhanced by inflammation.

Immunomodulation by cartilage has been described before, but until now the focus of* in vitro* studies had been on the ability of CHDR to synthesize specific cytokines such as IL-1*β* [2], bFGF, or aggrecan [4]. In addition, negative feedback reactions were described for human articular CHDR attenuating inflammation by secreting soluble IL-1RA (sIL-1RA) in response to IL-1*β* and IL-6 [10]. This effect was induced by increased transcription from the sIL-1RA promoter. This mechanism was elucidated further, showing that IL-1*β* function in CHDR is inhibited by a peroxisome proliferator-activated receptor *α* pathway which functions by an increase in sIL-1RA production [11]. The inflammatory stimulus applied in the used* in vitro* model is mediated not only by inflammatory cytokines produced by MNC, but also directly via Toll-like receptors present on human articular CHDR [12]. There is evidence that the suppressive effects of LPS on cartilage biosynthetic activity depend on the presence of TLR-4 and that the activation of this receptor is associated with IL-1*β* mRNA induction. The antianabolic effects mediated by TLR-4 in CHDR may play a role in acute infection and may be antagonized by BMP-7 [4, 12]. It is known that IL-1RA prevents interaction between IL-1 and its cell-surface receptors, thus competitively inhibiting the biological effects of IL-1 [13], but this does not fully explain anti-inflammatory effects of cartilage (mirrored by the tendency to reveal lower systemic CRP-levels in our patients with intact joints). Therefore, we aimed to investigate whether other anti-inflammatory mechanisms may be involved as upregulation of IL-4 and IL-10, as was recently suggested in association with treatment of acute lung injury or osteoarthritis [14, 15]. Our data support that both cytokines are expressed* in vivo* and* in vitro*, but neither of these proteins revealed an association with the presence or absence of cartilage. Recent studies have focused on FasL expression as another potential candidate for cartilage's anti-inflammatory effects. FasL expression by CHDR has been described as a possible mechanism to induce apoptosis in macrophages, thereby reducing the inflammation caused by cartilage grafts [16]. Furthermore, increased Fas and FasL levels were observed in the synovial fluid of patients with rheumatoid arthritis (RA) [17]. Data from that study [17] suggested that synovium modulates inflammation and proliferation via this molecular mechanism. Our data confirm this, displaying FasL expression in the coculture and in joints with and without CHDR. One can only speculate about the reasons for significantly lower concentrations in the coculture with CHDR, but CHDR might be acting as a scavenger rather than an active producer in this setup. This hypothesis is supported by the fact that SFB did demonstrate higher viability during infection in the presence of CHDR. Although the metabolic activity of CHDR during the coculture decreased slightly over time, the influence of inflammatory conditions was less than that shown in conjunction with SFB, suggesting compensating mechanisms that result in greater resistance. Furthermore, the attenuation of inflammation by CHDR, for example, via sIL-1RA secretion, may also be the reason for less FasL secretion in the coculture medium.

As shown previously, the secretion of inflammatory cytokines as IL-1*β*, monocyte chemoattractant protein- (MCP-) 1, and IL-8 by chondrocytes depends on their maturity and is inversely regulated to TGF*β* expression [18]. Toll-like receptors are expressed by mature chondrocytes, react to danger signals by production of further proinflammatory cytokines, and, therefore, support an inflammatory-driven cartilage degradation in a vicious circle [19]. The antagonistic effects of TGF*β* and IL-1 are mediated by regulation of TGF*β* receptor type II and Smad7 [20] and are similar to the relation of proinflammatory TNF*α* and BMP-2, a member of the TGF*β* superfamily [21]. Therefore, we hypothesized that TGF*β* might also mediate the immune-regulatory and anti-inflammatory effect of chondrocytes. Although we have not directly demonstrated this, we were able to show induction of TGF*β* mRNA in SFB by CHDR and increased TGF*β* levels in the coculture of SFB and CHDR supporting a regulatory association.

Aggrecan has been identified as a reliable indicator for OA progression and matrix degradation [22] that is upregulated following injury or general inflammation. Both the breakdown of extracellular matrix (ECM) and enhanced synovial secretion are known to cause this effect [23, 24]. The experimental data provided in this study confirm that both joint compartments, the synovium and cartilage, contribute to the aggrecan level increase we observed. Although the difference observed in the clinical trial between joints with and without cartilage did not reach statistical significance, we did note a tendency towards higher concentrations in the joints without arthroplasty. Furthermore, aggrecan levels rose steadily during the coculture in both groups, reaching higher concentrations in the presence of CHDR. Our understanding of the aggrecan's role has grown recently because it is not just an essential element of the ECM; it is also directly involved in immunoregulatory processes such as the induction of inflammation by HLA-B27-restricted epitopes [25].

bFGF (FGF-2) has been characterized as a chondrogenic mitogen typically involved in cartilage repair processes [5] demonstrating high potential in cartilage engineering [26]. Acute infection leads to increased synovial bFGF levels, but mRNA induction in CHDR is 10-fold higher than in the SFB [4]. bFGF is secreted by CHDR released from ECM, enhances CHDR proliferation, induces a chondrogenic phenotype dependent on the experimental setting, and promotes articular cartilage repair by upregulating multiple growth factors such as BMPs and TGF*β* [27]. Our data confirm this by demonstrating bFGF in the cavity of inflamed joints and gradually increasing concentrations in the* in vitro* model. Moreover, our results indicate that the potential of SFB to produce bFGF is remarkable and triggered by proinflammatory conditions. However, our clinical trial's data support that the main* in vivo* source is the cartilage itself. The time-dependent regulation pattern seen* in vitro* might be explained by the fact that bFGF is secreted by CHDR but may also be bound via receptors.

A critical evaluation of the presented* in vivo* data includes a comparison of both patient collectives. Naturally, they differ in the main discrimination criterion, the presence of an endoprosthesis, but they also had the same kind of clinical symptoms and in the subgroup analysis also the same effected joint with the predominantly same sort of causing bacteria. The groups differed in age, but based on a twin study no correlation between age and IL-1*β* expression could be found [28]. The same study reported a moderate heritability for IL-1*β* expression that declined with age due to an increase in unique environmental factors. Since the investigated population was rather old compared to the data published, the factor of heritability may be estimated to have a low influence. Therefore, both groups can be compared regarding the influence of the cartilage presence. Increased synovial IL-1*β* values should correlate with higher systemic inflammatory parameters. Despite not reaching statistical significance, the trend could be confirmed for leucocyte counts and plasma CRP-levels. This also plausibly explains the longer stay at an ICU, which really was statistically significant. Assuming a random distribution of other (unknown) parameters, a comparison based on the main discrimination criterion seems to be justified.

To summarize, we confirmed the hypothesis of cartilage's immunomodulatory properties. Acute infections induce processes that resemble OA development such as ECM degradation, the induction of cartilage repair cytokines, and the initiation of apoptosis. Generally speaking, cartilage would seem to exert a protective, anti-inflammatory effect that is at least partially associated with FasL action, resulting in the enhanced survival of SFB.

## Figures and Tables

**Table 1:** Comparison of patients with arthroplasty and normal joints using epidemiological and inflammatory parameters. The first data set shows the comparisons for all patients, the second for the subgroup of patients with exclusive knee infections  and knee arthroplasties. m: male, f: female, ASA: physical status according to the American Society of Anesthesiologists, ICU: intensive care unit, y: yes, n: no, and CRP: C-reactive protein in serum. ^*∗*^Aspin-Welch test, ^+^Chi square test, ^#^ *U*-test (Mann-Whitney), and ^++^ *t*-test.

	Endoprosthesis (*n* = 8)	Normal joint (*n* = 68)	*p*
All patients			
Age (years)	71.3 ± 3.4	58.3 ± 2.8	0.001^*∗*^
Gender (m/f)	7/1	44/24	n.s.^+^
ASA classification	3.0 ± 0.2	2.2 ± 0.1	0.01^#^
Body mass index	31.6 ± 1.6	27.3 ± 0.8	0.03^#^
ICU treatment (y/n)	6/2	16/52	0.002^+^
Initial leucocyte count (Tsd/*µ*L)	13.7 ± 1.9	10.9 ± 0.6	n.s.^++^
Initial CRP (mg/L)	154.9 ± 38.1	112.2 ± 10.8	n.s.^++^

	Endoprosthesis (*n* = 6)	Normal joint (*n* = 51)	*p*

Subgroup with exclusive knee infections			
Age (years)	69.1 ± 4.2	55.9 ± 3.3	n.s.^*∗*^
Gender (m/f)	5/1	37/14	n.s.^+^
ASA classification	3.0 ± 0.3	2.1 ± 0.1	0.02^#^
Body mass index	31.6 ± 2.1	27.1 ± 0.9	n.s.^#^
ICU treatment (y/n)	4/2	12/39	0.026^+^
Initial leucocyte count (Tsd/*µ*L)	13.0 ± 2.5	10.5 ± 0.7	n.s.^++^
Initial CRP (mg/L)	151.6 ± 52.9	103.0 ± 11.9	n.s.^++^
